# Supplementary material for: Are carbon emissions trading and green financial instruments synergistic? -Comprehensive quantitative research based on content analysis
Source: PLoS One. 2024 Mar 7;19(3):e0298601. doi: 10.1371/journal.pone.0298601 (PMC10919601; doi:10.1371/journal.pone.0298601)
Supplement: S1 Data — (ZIP) [file pone.0298601.s001.zip › Data/地级市Z_F工作报告.zip!/║╙─╧/╨┬╧τ╩╨/└┤╫╘╣1⁄2╓┌║┼í╛┬φ┐╦╩2╛▌═°í┐_2018.docx]

各位代表：

　　现在，我代表市人民政府，向大会作工作报告，请予审议，并请各位政协委员和列席会议的同志提出意见。

　　一、本届政府工作回顾

　　2014年以来，本届政府在省委、省政府和市委的坚强领导下，在市人大、市政协的监督支持下，以习近平新时代中国特色社会主义思想为指导，树牢“四个意识”，坚定“两个维护”，主动适应把握引领经济发展新常态，坚持稳中求进工作总基调，全力稳增长、促改革、调结构、惠民生、防风险，经济社会发展稳中有进、进中提质，全面小康迈出坚实步伐。

　　——综合实力大幅提升。地区生产总值年均增长7.9%，2017年（下同）达到2384.8亿元；一般公共预算收入年均增长5.3%，达到159.1亿元；规模以上工业增加值年均增长8.8%；社会消费品零售总额年均增长11.9%；固定资产投资年均增长10.1%；年粮食产量保持在80亿斤以上。成功争取郑洛新国家自主创新示范区、“中国制造2025”试点示范城市、国家产业集聚集群创新发展综合改革试点市、循环经济示范市、首批装配式建筑示范城市等一批国家级战略支持平台；全省首家军民融合产业基地、首个智能制造公共实训基地落户我市。

　　——发展活力不断增强。截至2017年年底（下同），累计实际利用外资33.7亿美元、省外资金2108.8亿元，年均分别增长7.7%、7.9%，口岸设施建设体系不断完善。各类市场主体从13.6万家增长到22.1万家。科研经费占地区生产总值比重、专利申请量、承担省级重大科技专项数量持续位居全省前列。

　　——产业结构持续优化。三次产业结构由12:56.6:31.4调整到9.5:49:41.5，工业增加值、服务业增加值对经济增长的贡献率分别达到43.8%和43.3%，我市经济由过去的工业主导，演变为工业和服务业“双轮驱动”格局。白鹭化纤、心连心化肥、科隆集团等龙头企业茁壮成长。“互联网+”、大数据、电子商务等新业态新模式蓬勃发展。

　　——基础能力得到加强。城镇化率达到51.96%，高于全省1.8个百分点。成功创建全国文明城市，通过国家园林城市、国家卫生城市复审，新乡的魅力与颜值俱增。郑新融合上升为全省战略，郑济高铁新乡段、G107东移改线、新日高速封丘至原阳段、沿太行高速新乡段开工建设，现代交通体系日趋完善。对接全省“三区一群”等战略，推动“三带三区两城十镇”战略布局，中心城区辐射带动能力增强，唐庄、孟庄等14个镇入选全国重点镇，城乡面貌持续改善。

　　——生态环境大幅好转。“三个体系、三个机制”成为加强生态文明建设的制度保障。PM10、PM2.5平均浓度分别较2014年下降15.3%、18.5%，优良天数由177天增加至222天，在2017年全省大气污染防治工作综合考核中排名第一。7个国控、省控地表水断面全面达标。我们用不足两年的时间，实现了环保工作从被约谈到受表彰的艰难逆袭，谱写了大气污染治理新篇章。

　　——人民生活明显改善。财政民生支出累计达975.4亿元，年均增长10.2%。18.94万贫困群众实现脱贫。城镇累计新增就业超过37万人，新增农村劳动力转移就业超过13万人。城镇非私营单位在岗职工年平均工资达到48886元，是2013年的1.46倍。9个县（市）、区通过国家义务教育均衡县验收。社会保险实现法定人群全覆盖，企业退休人员养老金提高33.9%，居民基础养老金最低标准、城乡居民基本医疗保险补助标准分别提高33.3%和60.7%。先后5次提高低保、五保救助标准，城乡低保标准分别提高34%和64%，特困人员集中供养、分散供养标准分别提高97%和94%。城镇居民、农村居民人均可支配收入分别达到29070元和13769元，是2013年的1.3倍和1.4倍。

　　本届政府主要做了以下工作：

　　（一）全力稳定经济运行。稳定实体经济。完善各级领导分包联系企业服务长效机制，落实降成本各项措施，最大限度减免企业税收。实施质量强市战略，中国驰名商标由9件增加到21件。扩大有效投资。抓好重大项目建设，累计开工亿元以上项目855个，完成投资3246.8亿元，华兰生物单抗生产基地、中纺院绿色纤维、平煤国能固态锂离子电池、中兴智慧产业园、新乡大数据产业园、中国电波科技城、氢能燃料电池研发中心等重大项目实施。防范化解风险。持续优化金融环境，金融机构存、贷款余额分别达到2541.3亿元和1503.7亿元，是2013年末的1.5倍和1.4倍。新三板挂牌企业达到37家。依法打击处置非法集资活动，化解担保链风险，银行不良贷款率持续下降，守住了不发生系统性、区域性风险的底线。

　　（二）加快推进转型升级。推进产业转型攻坚。突出抓好先进制造业专业园区建设、大企业（集团）培育，10个专业园区规划全部完成，建成“一台两器”18个，20家大企业（集团）在建、新开工项目63个，完成投资133.4亿元。推进“三去一降一补”，2016年以来，退出煤炭产能15万吨，淘汰关停水泥产能312万吨，全部取缔“地条钢”制售企业。推进农业提质增效。“四优四化”建设成效初显，优质小麦面积97万亩，经济作物面积295万亩，标准化养殖场由319家增加到518家。销售收入10亿元以上的农业产业化集群18个，国家级农产品品牌94个。推进服务业转型。旅游业接待游客年均增长12.9%，总收入年均增长15.8%，辉县市获批国家级旅游业改革创新先行区。金融业增加值增长50.6%。电商企业达到254家。

　　（三）持续加快动能转换。深化“放管服”改革，行政审批事项由199项精简至110项，清理规范中介服务事项276项。推进国企改革攻坚，圆满完成驻新省属企业剥离办社会职能工作。基本完成农村土地承包经营权确权登记颁证。完成县级农信社改制组建农商行工作。城市公立医院全面取消药品加成。城市管理、投融资等体制改革稳步推进。加强“一招四引”，与新松机器人、华为集团、平煤神马、台湾友嘉实业等一批知名企业建立合作关系，一批项目正在实施。建立跨境电子商务“单一窗口”综合服务平台，引进意大利欧玛控股等一批外向型企业。完善创新创业激励政策，构建“N＋1”科技创新政策体系。在全省率先推进自创区体制机制改革，高新区、经开区完成了“全员聘任制、绩效考核制、薪酬激励制”改革。大力培育创新引领型企业、人才、平台和机构，新增高新技术企业59家，省级以上研发中心达到279家。培养高技能人才4.2万人，获得国家科学技术二等奖以上奖励8项。万人发明专利拥有量达3.35件，位居全省前列。

　　（四）加快推进城乡建设。推进中心城区建设。严格规划管控，编制完成水系景观设计、海绵城市建设等60项规划。加快基础设施扩容提质，新建改造城市道路36条、污雨水泵站21座、供水管网49.1公里、供气管网425公里，新增各类绿地269.2公顷。在建棚户区和城中村改造项目50个，完成拆迁890万平方米。实施“温暖新乡守护蓝天”工程，入选北方地区冬季清洁取暖试点城市，市区供热普及率由36%提高到85.4%。实施百城建设提质工程。开工项目177个，完成投资35.4亿元，基础设施保障能力显著增强。长垣县成功创建全国县级文明城市。推动美丽乡村建设。整治农村人居环境，改善农村基础设施，解决72.4万农村居民和师生饮水安全问题，新建改建农村公路1685公里，95%的村庄建立了卫生长效保洁机制。

　　（五）持续保障和改善民生。推进精准脱贫。围绕精准扶贫、精准脱贫，扎实开展驻村帮扶，抓好产业扶贫、金融扶贫、易地扶贫搬迁、转移就业等关键环节，强化教育、交通、医疗、水利等行业扶贫，完成省定减贫目标。350个贫困村退出贫困序列，13525名贫困群众实现易地搬迁，滩区迁建试点工作得到中央和省委、省政府的充分肯定。治理环境污染。将环保作为必须守住的政治底线、发展底线和民生底线，大力实施蓝天、碧水、净土工程，生态环境持续改善。发展公共事业。城镇登记失业率控制在4.5%以内。全面改善1223所农村薄弱学校办学条件；新建、改扩建城镇中小学校69所、幼儿园280所，新增学位4.7万个；进城务工人员随迁子女就学实现应入尽入、同城同待遇。免费为城乡居民提供15类57项基本公共卫生服务，居民基本医疗保险实现城乡统一，参保率稳定在99%以上。分配保障房5.7万套，改造农村危房1.8万户。养老床位数达到1.8万张。加快公共文化四级服务网络建设，城区综合文化服务中心实现全覆盖。共享单车在中心城区投放。全面二孩政策平稳实施。加强社会治理创新。食品药品安全监管全面加强，生产安全事故起数和死亡人数分别较2013年下降62%和9.5%。探索推进诉调对接多元化解机制，化解信访积案，维护群众合法权益。荣获全国双拥模范城“六连冠”。依法依规抓好企业改制、问题楼盘等疑难复杂问题化解，确保国家和群众利益不受损失。严厉打击各类违法犯罪活动，群众安全感持续提升。

　　2018年以来，本届政府深入贯彻省委、省政府和市委的决策部署，实施“六个抓手”，即以“中国制造2025”试点示范城市建设为抓手，加快产业结构优化升级；以郑洛新国家自主创新示范区建设为抓手，深入推进创新驱动发展；以郑新深度融合为抓手，优化城乡结构，加快推进新型城镇化；以加快新旧动能转换为抓手，持续深化改革开放；以“两县十乡（镇）百村”示范工程为抓手，深入实施乡村振兴战略；以落实三年行动计划为抓手，打好打赢三大攻坚战，切实抓好新能源电池、电子信息、现代纺织、装配式建筑、文旅康养、生物育种“六大专项”，着力构建“六大体系”，经济社会持续向好。一是经济平稳增长。上半年完成地区生产总值1194亿元，同比增长8%。前8个月，规模以上工业增加值增长8.3%，居全省第2位；固定资产投资增长11.3%，居全省第1位；一般公共预算收入120.7亿元，居全省第3位，增长12.1%。二是后劲持续增强。鲁花食品产业园、纳米空心硅及硅基材料产业园、三一集团装配式建筑等重大项目落户新乡，新飞电器正式复产，新松机器人产业园、新乡过滤产业园、克明面业五谷道场、凤泉湖引黄调蓄及配套工程开工建设。三是基础不断夯实。全力打好“三大攻坚战”，非法集资案件高发势头得到遏制，产业扶贫和增收措施覆盖率达到100%，大气主要污染物浓度持续下降，卫河、共渠流域水环境治理全面启动，高质量发展的思路更清、根基更稳、步伐更快。

　　本届政府自觉接受市人大法律监督和市政协民主监督，积极听取各民主党派、工商联、无党派人士和人民团体意见，认真办理人大议案和政协提案。严格落实中央八项规定精神，弛而不息整治“四风”，“三公”经费下降75%，反腐倡廉工作取得新进展，政府自身建设和治理能力不断提升。民族宗教、人民防空、统计审计、新闻出版、外事侨务、史志档案、气象地震等工作都取得新成绩。

　　各位代表！这些成绩的取得，是我们认真践行习近平新时代中国特色社会主义思想的结果，是省委、省政府和市委正确领导的结果，是全市人民团结拼搏的结果。在此，我代表市人民政府，向所有关心、支持、参与新乡发展的同志们、朋友们，表示衷心的感谢并致以崇高的敬意！

　　各位代表！这四年多时间是新乡发展进程中极不寻常、浴火重生的时期，我们观势、借势、驭势，围绕转型发展，加快改革创新，发展动能持续增强，把握了发展的主动权；我们全力争取国家级战略支持平台，为发展赢得了广阔空间；我们大力开展环境治理，扎实推进脱贫攻坚，群众幸福感不断提升。四年多来，还有许多往事令人难忘，全市人民万众一心、众志成城，战胜了2016年“7·9”“7·19”两场特大暴雨洪涝灾害，实现了环境污染防治的绝地反击，处理了新晋高速等一批重大历史遗留问题。这些都让我们坚信，只要一切依靠群众、一切为了群众、一切服务群众，凝心聚力、握指成拳，就一定能够战胜前进道路上的任何艰难险阻。

　　各位代表！本届政府在工作中有几点深刻体会：必须牢固树立“四个意识”。党的十八大以来，习近平总书记举旗定向、把舵领航，成功驾驭我国经济社会发展大局，在统揽“四个伟大”实践基础上形成的习近平新时代中国特色社会主义思想，为我们战胜各种风险挑战、推动经济社会发展指明了前进方向，是我们取得各项成绩的根本所在。必须融入大局。我们坚持在大势大局中兼顾当前和长远，着力打好“四张牌”，谋划实施新时代新乡的创新体系、产业体系、城镇体系、生态体系、基础能力支撑体系、社会保障和公共服务体系及郑新融合、先进制造业专业园区、“三带三区两城十镇”等工作，努力使之成为破解新乡发展难题的“金钥匙”。必须突出特色。在新常态下，必须找准并充分发挥自身比较优势，持续抓重点、解难点、举亮点，塑造城市个性，擦亮城市品牌，才能在区域融合、专业化分工中做大做强，打造竞争新优势。必须保持定力。始终直面矛盾、勇于担当，在实践证明正确的路子上踏石留印，在行之有效的举措上抓铁有痕，一张蓝图绘到底、风吹浪打不动摇、不达目的不罢休。必须精准发力。在新常态下，经济发展进入精准发力时代，必须尊重自然规律、经济规律、社会发展规律，以精准的举措、科学的方法推进工作，才能达到四两拨千斤的效果。这些既是我们积累的经验，也是下一步工作的着力点。

　　各位代表！我们也清醒地看到存在的问题和不足：一是县域经济发展水平不高。城乡之间、县区之间发展水平和质量差异较大，短板弱项突出，制约全市经济高质、高速发展。二是增长动力不足。传统产业占比偏重，龙头企业数量偏少，低端低质低效问题仍较突出，体制机制不活，经济外向度低，创新能力亟待加强。三是风险隐患较多。企业经营风险、金融风险、社会领域风险不容忽视。四是民生欠账较多。脱贫攻坚任务艰巨，环境压力依然较大，城乡居民收入偏低，群众对住房、就业、教育、医疗等方面还有不少不满意的地方。五是自身建设有待加强。职能转变不到位，工作人员能力素质还不适应新时代要求，一些领域的不正之风和腐败问题依然存在。我们将在今后工作中努力解决这些问题。

　　二、今后五年的目标任务

　　当前，我国经济发展进入新时代，由高速增长阶段转向高质量发展阶段。对我市而言，一方面，既面临加快转型、提高发展质量的共性要求，也面临扩大总量、补齐短板等艰巨任务，习近平总书记指出的跨越非常规的我国经济发展现阶段特有的关口和跨越常规性的长期性的关口在我市表现得尤为突出。另一方面，要看到当前世界经济复苏回暖，我国经济稳中向好，市场活力迸发，这些都将为我市经济稳定运行提供有利的宏观环境。特别是近年来，我们在工作布局上明确了方向，在绿色发展上争取了主动，发展的势能正在集聚、空间不断拓展、环境持续优化，只要我们紧盯目标，持之以恒，埋头苦干，一定能够迎来美好的明天。

　　今后五年的总体要求是：以习近平新时代中国特色社会主义思想为指导，全面贯彻党的十九大精神，坚持稳中求进工作总基调，坚持新发展理念，主动适应社会主要矛盾变化要求，统筹推进“五位一体”总体布局和协调推进“四个全面”战略布局，坚持高质量发展根本方向，坚持以供给侧结构性改革为主线，统筹稳增长、促改革、调结构、惠民生、防风险，着力发挥优势打好“四张牌”，着力打好“三大攻坚战”，着力实施产业强市、创新驱动、开放带动、区域协调发展、乡村振兴“五大战略”，着力突出“六个抓手”、抓好“六大专项”、构建“六大体系”，着力深化改革开放创新，促进经济社会持续健康发展，决胜全面建成小康社会，开启新时代新乡全面建设社会主义现代化新征程，在谱写中原更加出彩新篇章中发挥更大作用、作出更大贡献。

　　今后五年主要经济指标年均增速高于全省平均水平，力争人均指标达到全省平均水平，县域经济实力在全省位次不断前移，“六大体系”建设取得新成效，高质量发展取得新进展。

　　一要着力构建现代创新体系。依托郑洛新国家自主创新示范区平台优势，推进“一区四园四集群”建设，聚焦“四个一批”，集聚创新要素，培育创新平台，深化体制机制改革，打通政产学研金深度融合、军民企地对接转化、市场运作、人才成长等通道，改善创新创业环境，强化知识产权创造、保护和运用，持续增强科技实力和创新能力。二要着力构建现代产业体系。紧盯产业链的终端、价值链的高端、创新链的前端，以供给侧结构性改革为方向，突出抓好四大传统产业优化升级、四大支柱产业提质增效、四大战略性新兴产业加速发展，加快以“一台两器一园”为主体模式的先进制造业专业园区建设，培育大企业（集团），推进“三大改造”，增强要素保障能力，推动现代服务业、特色高效农业与先进制造业协同发展。三要着力构建现代城镇体系。坚持“向心布局、组团融合、轴带发展、节点提升”的总思路，充分发挥中心城市的辐射作用、县城的带动作用和特色城镇的基础作用，以自然资源、文化资源、产业配套为支撑，高水平推进“三带三区两城十镇”建设，破解空间要素制约，完善城镇产业、服务、研发、居住四大功能。四要着力构建生态保护体系。坚持绿色发展理念，实施蓝天、碧水、净土工程，加强“32231”为主的林业生态和“五湖四河两渠”为主的水生态体系建设，促进生产空间集约高效、生活空间宜居适度、生态空间山清水秀。五要着力构建基础能力支撑体系。坚持全面推进与重点突破相结合，按照网络化布局、智能化管理、一体化服务的导向，大力推进交通、水利、信息、物流等基础设施建设，完善铁、公、机、海和场站枢纽在内的外联内通综合交通网络。六要着力构建社会保障和公共服务体系。坚持民生优先，不断满足人民日益增长的美好生活需要，多层次社会保障体系更加完善，教育和医疗卫生水平不断提升，现行标准下农村贫困人口全部脱贫，区域性整体贫困有效解决；社会治理更加有效，文明程度显著提高，政务环境更加廉洁高效，彰显公平正义、共建共治共享的社会治理格局基本形成。

　　工作中要始终坚持以战略思维谋全局、以辩证思维破难题、以法治思维图善治、以底线思维防风险、以民本思维惠民生，着力把握好以下原则：坚持党对一切工作的领导。树牢“四个意识”，坚定“两个维护”，全面加强党的政治建设，不断提高干部队伍的凝聚力和战斗力。坚持以人民为中心的发展思想。把人民对美好生活的向往作为奋斗目标，在“七个有所”上更多用力，让人民群众有更多获得感、幸福感、安全感。坚持稳中求进工作总基调。正确处理好稳增长与促改革、调结构的关系，把握好工作节奏和力度，做到调速不减势、量增质更优。坚持推动高质量发展。把转型发展作为主攻方向，持续推进供给侧结构性改革，重点抓好决胜全面建成小康社会三大攻坚战。坚持统筹兼顾的工作方法。既要坚定不移推动高质量发展，又要在确保质量效益的前提下，加快发展速度；既要坚持重点突破，又要统筹各项事业协调发展。坚持深化改革开放创新。完善体制机制，适应矛盾新变化、发展新阶段、科技新进步、人民新需要，在新的历史起点上，思想再解放、改革再深化、开放再扩大。坚持把项目建设作为总抓手。将各项目标落实到项目上，拉清单、立专案、建台账、明责任、设时限、定奖惩，确保工作落到实处。

　　三、2018年重点工作

　　今年经济社会发展主要预期目标是：地区生产总值增长8%，全社会固定资产投资增长9%，社会消费品零售总额增长11%，进出口总值稳步增长；第一二三产业分别增长3.5%、8%、9%以上，第三产业增加值占比进一步提高，规模以上工业增加值增长8%以上，高技术产业增加值占规模以上工业增加值比重达13%，常住人口城镇化率提高1.5个百分点以上；一般公共预算收入增长8%，税占比达70%以上，研究与试验发展经费投入强度达2.1%，节能减排、环境保护等约束性指标完成省下达任务；城镇调查失业率和登记失业率分别控制在5.5%和4.5%以内，居民人均可支配收入实现与经济增长同步，居民消费价格涨幅3%左右。

　　当前距年底仅剩3个月时间，我们将围绕圆满完成全年各项目标任务，强力攻坚，全力冲刺。

　　（一）持续壮大发展规模。把发展的着力点放在实体经济上，坚持优化存量与扩充增量并举，推进经济发展行稳致远。优化存量。把现有企业发展壮大作为一项重大战略推进，对产品有市场、有效益、企业有条件的支持其扩大规模、做大做强。持续深化企业服务，强化精准调度，加强对投资、用电量、税收、订单等指标的动态监测。支持民营企业发展，把民资当外资、把老乡当老外，一视同仁、同等对待。推进企业家培养计划，弘扬企业家精神和工匠精神，营造尊重、鼓励、保护企业家干事创业的社会氛围。大力简政减税减费，降低企业制度性交易成本和生产经营成本。实施质量强市战略，培育、提升一批知名品牌。扩充增量。完善周调度、月集中开工、月讲评、季观摩等机制，集中精力抓好一批“双百双五”“双十双五”项目。全年新开工230个亿元以上项目，白鹭化纤1万吨纤维素长丝等200个亿元以上项目竣工投产。

　　（二）加快推进高质量发展。坚持质量第一、效益优先，举龙头、立支柱、铸品牌、强载体，持续提高产业高端化、绿色化、智能化、融合化水平，推进经济发展质量变革、效率变革、动力变革。

　　一是推动产品结构升级。加快先进制造业专业园区建设。突出先进、智造、专业发展定位，以“一台两器一园”为主体模式，完善基础设施，打造产业集聚区的升级版。建立“一个牌子、一个机构、一套班子、一套平台、一套机制”的管理模式和指标统计体系。加快中融航电卫星导航工程技术中心、华兰生物单抗研究中心等平台建设。推进中国电波科技城、“中国制造2025”原阳示范区、新乡机器人产业园等园区建设，形成能进能出、竞相发展的良好局面。实施重大示范专项工程。推进与清华大学、同济大学的合作，加快平煤国能固态锂离子电池项目建设，大力引进整车企业，推进动力电池与新能源汽车产业发展。加快中纺院绿色纤维基地建设，扩大高档化纤产业规模，引进终端品牌龙头企业，推进绿色纤维及纺织服装产业发展。加强政策引导，支持绿色装配式建筑产业发展。创建“中国制造2025”国家级示范区。实施增品种、提品质、创品牌“三品”专项行动，推进智能制造、绿色制造、精益制造、品牌制造，支持企业发展个性定制、高端定制。推进制造业绿色化改造、智能化改造和技术改造，优化提升装备制造、食品加工、纺织服装、现代家居等支柱产业，培育动力电池与新能源汽车、生物与新医药、电子信息、航空航天等战略性新兴产业。推动产业集聚区晋位升级，完成固定资产投资1100亿元，实现主营业务收入2700亿元。推进军民融合发展。建设“一基地三园区”，实施平原航空机电大飞机产业园等21个总投资161.5亿元的军民融合项目。

　　二是推动组织结构升级。培育大企业（集团）。支持白鹭化纤、心连心、新航集团、科隆集团、银金达等企业发展壮大，力争主营业务收入超百亿元的企业达到3家、超50亿元的企业达到4家。支持康佳集团做好新飞电器生产经营和研发能力建设，力争通过三年时间，新飞冰箱重回国内冰箱行业一线阵营。推动华兰生物争创国内一流新型医药产业基地。坚持引进与培育相结合，紧盯与中国通用、中航工业、中兴通讯、友嘉实业、奥克斯等企业的合作，承接产业转移。扶持中小企业发展。加快个转企、小升规、规改股、股上市工作，分层次、分行业、分梯队建立企业升级后备资源库，形成大中小企业梯次推进、协调发展的格局。鼓励企业进入全球产业链、价值链、创新链、供应链，优化企业资金链，打造促进发展的政策链，推动“六链”系统融合和统筹协调。

　　三是推动技术结构升级。加快自创区建设。加强与知名高校、科研院所和大企业（集团）的合作，在高新区打造动力电池与新能源汽车、生命科学和生物工程、大数据、航空航天军民融合“四大产业集群”。推进新松机器人一平台多基地、中国小麦产业研究院、863科技产业园、中纺院中原分院、中电科18所新乡电池检测中心、新乡创新谷等平台建设，加快氢能燃料电池、3D打印产业园、华大基因、九次方大数据、华为大数据产业园等项目建设。提升创新能力。推进自主创新、开放创新、协同创新，新培育高新技术企业35家、科技型中小企业40家、省级以上科技创新平台25家，各类研发平台总量突破700家，孵化载体达25家以上。整合驻新高校和科研院所创新资源，举办第一届高校院所河南科技成果博览会，打造我市开放创新的靓丽名片。创新体制机制。深化自创区管理体制和人事薪酬改革，推进高新区、经开区扩区。推动科技与产业、科技与金融、产业与金融、军工与民用融合。深化科技金融产品和服务创新，扩大“科技贷”规模，培育引进各类创新创业基金。大力实施“牧野英才”“名校英才”“牧野工匠”计划，完善吸引、留住、用好人才的政策措施，为未来赋能。

　　四是推进服务业转型升级。顺应居民消费结构向高端化、个性化、多样化方向升级趋势，加快健康、旅游、文化、金融、物流、电子商务、大数据等现代服务业发展。引进国内外知名机构和企业在我市建立区域性总部、分支机构、研发中心、销售结算中心。支持辉县市创建国家全域旅游示范区，建设国家级旅游业改革创新先行区，确保八里沟成功创建5A级景区。巩固农信社改革成果，推进市级农商行组建，做强地方金融。加快高铁物流、电商物流、冷链物流发展。支持企业与华为等主要云服务提供商合作，打造“云上”产业发展载体。

　　（三）推进区域协调发展。以“三带三区两城十镇”为抓手，以郑新深度融合为重点，大力推进郑新一体化进程，统筹区域融合、城乡融合、产业融合，加快建设新型城镇体系。

　　一是打造重要经济带。以产业为基础，推进郑新产业带、沿黄生态景观产业带、南太行文旅康养产业带“三带”和郑洛新国家自主创新示范区、“中国制造2025”国家级示范区、中国（河南）自贸区新乡辐射区“三区”建设。打造郑新产业带。按照“规划引领、交通先行、产业协同、功能互补、节点突破、统筹推进”的思路，推进交通互联、产业互融、功能互补、生态共建等领域重大项目建设。以郑济高铁、郑新快速通道等基础设施互联互通为依托，加快郑新融合交通廊道建设。积极承接郑州资本有机构成较低的绿色产业转移。建立健全跨区域生态文明建设联动机制，推进全方位融合。打造沿黄生态景观产业带。围绕扮靓母亲河，建设沿黄生态景观带，推进中部植物园、林业科创小镇等项目实施。以平原示范区、原阳县产业集聚区、封丘县产业集聚区、获嘉县华夏幸福产业新城等为战略支点，建设沿黄产业带，加快与郑州的产业协同，努力使景观与产业共生共融、相得益彰。打造南太行文旅康养产业带。立足妆点太行山，建设南太行生态景观带，做好太行山区森林资源培育和保护工作，发展特色生态旅游和健康养生养老产业，形成郑州组合型大都市地区的“后花园”。加快自贸区新乡辐射区建设。复制自贸区政策，推进经开区保税物流中心建设，引导制造业发展跨境贸易，推进优势产业货通全球。

　　二是打造关键支撑点。加快中心城区建设。坚持“东扩、西提、南融、北治”并举，编制我市2049（空间）发展战略规划，启动新一轮城市总体规划修编。加快部分路段和街区改造提升，推进示范景观街区建设。实施园林绿化提质等十大工程，打造金穗大道、平原路、宏力大道和新飞大道、和平大道、劳动街“三横三纵”的景观大道，完善科隆大道等36条城市道路及配套设施建设。抓好国际商务中心建设，推进飞机场搬迁。加快实施三年棚改攻坚计划，强力推进54个棚户区（城中村）改造。大力实施“守护蓝天温暖新乡”工程，集中供热普及率达到95%以上，2019年年底达到100%。持续抓好“五湖四河两渠”生态水系建设，加快凤泉湖建设，做好卫源湖、东湖前期工作。加快“两城”建设。抓好凤凰山和凤泉湖山水林田湖草一体化生态城规划设计，推进区域范围内空间立体性、平面协调性、风貌整体性、文脉延续性的规划管控，打造北方水乡、园林经典、文化圣地、旅游天堂、创新乐园、生态新城，使之成为中原城市群的一颗璀璨明珠。依托延津县3.7万亩国有林场资源，高端策划、科学规划，加快推进延津县主题森林城建设。抓好“十镇”建设。谋划建设陈桥宋源文化风情小镇、平原示范区原武特色小镇、辉县百泉古镇、宝泉文旅小镇、获嘉同盟古镇、卫辉古城、卫辉财神小镇、唐庄航空小镇及刘庄、小冀等特色小镇，助推乡村振兴。

　　三是深入推进百城建设提质。把百城建设提质与文明城市创建结合，突出以水“润”城、以绿“荫”城、以文“化”城、以业“兴”城、以法“治”城，推进文明城市创建常态化。抓好城市功能修补、生态修复、文化滋养和路网、林网、水网、管网“四网”建设，推进交通秩序治乱、市容卫生治脏、生态环境治污、公共服务治差工作，持续提升城市管理水平。加快农业转移人口市民化，让符合条件的农业转移人口在城市落户定居。

　　四是提升交通支撑能力。加快铁路建设。加快郑济高铁新乡段建设，推进新焦城际铁路前期工作。围绕“全互通、零换乘、不对流、无障碍”的目标，谋划高铁东站双面站建设，打造全省和郑州大都市区重要的铁路客货运枢纽。完善高速公路和干线公路网络。加快新延快速路、S229快速路、G107东移改线和新晋高速、新日高速封丘至原阳段、沿太行高速新乡段“三条公路、三条高速”建设。加快郑新快速路前期工作，力争年内具备开工条件。推进官渡黄河大桥、鹤辉高速、菏宝高速长垣段、黄河北堤堤顶道路改造等项目建设，积极开展安罗高速新乡段、兰原高速兰考至封丘段、郑新高速（新晋高速南延）、龙凤大道、G230新乡黄河大桥等项目前期工作。推进区域融合。加快推进县城与中心城区融合，谋划建设新辉、新新、新卫、新获、新原、新长等快速通道。围绕建好、管好、护好、运营好，持续抓好农村公路建设，新建、改建农村公路300公里以上。

　　（四）实施乡村振兴战略。大力推进乡村产业、人才、文化、生态、组织“五个振兴”，推动农业全面升级、农村全面进步、农民全面发展。

　　一是推进精准脱贫。坚持以脱贫攻坚统揽经济社会发展全局，完善责任、考核、督查巡查“三个体系”和问题排查、台账管理、暗访检查、跟踪督查、人员培训、奖惩激励“六个机制”，打好产业扶贫、就业创业、金融扶贫、生态扶贫“四大硬仗”，开展健康扶贫、教育扶贫、扶贫助残、易地扶贫搬迁、危房改造清零、扶贫扶志“六大行动”，实施交通设施建设、安全饮水、电网升级改造、农村环境整治“四大工程”，加大企业带贫困户、合作社带贫困户、专业大户带贫困户“三带”力度。注重扶贫与扶志扶智结合，统筹非贫困县、非贫困村精准脱贫工作，确保5.13万贫困人口稳定脱贫，封丘县、原阳县实现脱贫摘帽。加快滩区迁建和易地扶贫搬迁，第二批试点达到搬迁入住条件。

　　二是发展现代农业。推进结构转型。加强耕地质量保护，实施“藏粮于地”“藏粮于技”战略，增强粮食生产能力，保障国家粮食安全，确保粮食产量稳定在80亿斤以上。培育优质小麦、花生、草畜、林果等产业集群，优质小麦、优质花生种植面积分别达到100万亩以上，创建生态养殖示范场30家。提高绿色优质农产品供给，提升“中国第一麦”品牌效应。推进方式转变。抓好田园综合体试点建设，发展观光农业、都市农业、设施农业。推进信息技术在农业领域的应用，提高农业信息化水平。加快中农联农特产品批发市场、鲁花食品产业园等项目建设。推进动能转换。加强农业科技创新，推进河南省生物育种中心建设，依托中国农科院、省农科院两个基地，争创国家农业高新技术产业示范区。培育新型农业经营主体和职业农民，发展多种形式适度规模经营。支持农民返乡创业，鼓励各类人才到农业农村大显身手。

　　三是建设“四美乡村”。围绕环境美、田园美、村庄美、庭院美，推进农村人居环境整治。完善基础设施和公共服务，加快生活垃圾、污水处理和村容村貌美化，抓好“厕所革命”。实施“两县十乡（镇）百村”示范工程三年行动计划，完成110个行政村环境综合整治。加快南水北调配套工程建设。强化基层组织建设，健全自治、法治、德治相结合的乡村治理体系。解决好新型农村社区遗留问题。

　　（五）持续改善生态环境。牢固树立绿水青山就是金山银山的理念，加快构建森林、湿地、流域、农田、城市五大生态体系。深入实施蓝天工程。强化“六控”措施落实，确保空气质量持续改善，力争退出全国169个重点城市后20名。加快淘汰落后产能，推进清洁能源替代利用，取消建成区用煤。推进覆盖范围更广的污染物在线监控体系建设，防止已取缔企业死灰复燃。持续推进碧水工程。按照“全面清河、控源截污、达标治理、清淤疏浚、开源引水”的思路，推进全域治理、系统治理、源头治理，确保出境河流水质稳定达标。落实“河长制”，全部消除市区建成区黑臭水体，卫辉市、辉县市完成整治任务。加快建设新乡县等7座污水处理厂，完成南水北调二级保护区违法建筑和畜禽养殖场搬迁整治。编制卫河全域保护规划，推进卫河、共渠、东大沙河、东孟姜女河等清淤疏浚工程建设，加快市区斜沟清淤整治。完成辛庄、人民胜利渠渠首等引黄口门改造，推进西孟姜女河等4座节制闸除险加固工程、卫河下游新建节制闸建设。扎实推进净土工程。以土壤污染综合防治先行区建设为契机，聚焦重点区域、行业和污染物，开展土壤污染治理与修复。推进重金属污染物总量减排，加强重点污染源管控，绝不能增加新的污染。加强农村农业面源污染防治，实现秸秆、农膜、畜禽粪便等废弃物资源化、无害化利用。加强生态修复和保护。建立统一的空间规划体系和协调有序的国土开发保护格局，推进生态保护红线、永久基本农田、城镇开发边界三条控制线划定实施。加强地质灾害防治，狠抓矿山开采秩序整顿，加强矿山环境治理与修复。坚决整治河道非法采砂，恢复河流自然生态。实施“32231”林业生态工程，抓好沿黄、卫河、南太行三个生态景观带建设，加快凤凰山和凤泉湖山水林田湖草一体化生态城、延津县主题森林城建设，强化黄河湿地鸟类、太行山猕猴两个自然保护区管理提升，打造封丘、获嘉、凤泉（含牧野区王村镇）三个花卉苗木基地，建设好国家储备林基地，构筑生态发展屏障。完善环保治理体系。严格落实“三个体系、三个机制”，建立严格的源头严防、过程严管、后果严惩制度。完善生态文明建设考核机制，推进生态环境损害赔偿制度改革，健全信用评价、信息强制性披露等制度，强化排污者责任，构建政府主导、企业主体、社会组织和公众共同参与的环境治理体系。

　　（六）严控化解各类风险。强化底线思维，打好防范化解重大风险攻坚战。防范化解金融风险。突出降杠杆、治乱象、抓规范、强监管，持续打击非法集资、金融犯罪、网络传销。开展非法集资“案件处置兑付攻坚年”活动，最大限度挽回群众损失。综合利用资产管理、并购基金、不良贷款处置或回购等方式，加强与中原资产管理公司的合作，探索经验、总结方法、形成示范，逐步化解担保链风险。防范化解政府债务风险。开展政府债务审计，严格控制增量，稳妥化解存量。加强融资平台管理，新乡投资集团资产规模力争突破300亿元。树立经营城市理念，加强土地储备，增强自我增值、自我积累、自我投入、自我发展的能力。防控企业经营风险。利用政府可控资源，调动信贷投放的积极性，促进金融与实体经济良性循环。落实主办银行制度，发挥债权人委员会作用，对有市场、有效益、有信用、有技术的企业，不压贷、不抽贷、不断贷。引导企业制定金融战略、创新战略、品牌战略，优化资产结构、债务结构，力争把企业负债率降到合理水平。支持瑞丰新材、科隆新能源、山水环境、天力锂能等企业上市，推动企业融资由以债为主向以股为主转变。防控房地产风险。加强土地市场监测，促进房地产供需总量平衡和结构优化。建立多主体供应、多渠道保障、租购并举的住房制度，基本建成各类保障性住房1.3万套以上。坚持依法依规、先易后难，推进问题楼盘矛盾化解，全年解决问题楼盘存量70%以上。健全制度体系，防止产生更多的难了之案、难解之题。防控安全生产风险。严格落实地方党政领导干部安全生产责任制，全面做好治安、交通、消防等公共安全管理，强化安全隐患排查治理和专项整治，确保实现“一个杜绝、三个持续下降”目标。严厉打击食品药品违法行为，争创国家食品安全示范城市。

　　（七）持续推进改革开放。深化重点领域改革。按照党中央、国务院和省委、省政府的统一部署，稳步推进机构改革。持续打好国企改革攻坚战，全面完成国有“僵尸企业”处置工作，妥善解决债务处理、债权保护、资产处置、职工安置等问题；完成市属国有工业企业改革，做好“三供一业”分离移交等工作；推进企业产权结构、治理结构改革，建立现代企业制度；加强国有资产监管，加快由管企业为主向管资本为主转变。深化“放管服”改革，推进“三十五证合一”，全面实施市场准入负面清单制度，力争使项目审批时间再砍掉一半，政务服务一网办通，企业和群众办事力争只进一扇门、最多跑一次，凡是没有法律法规规定的证明一律取消，压缩企业开办时间，推进行政审批流程再造、全程监控、红灯报警、密切协同、明责问责，为市场主体添活力，为人民群众增便利。深化农村改革，落实好第二轮土地承包到期后再延长30年相关政策，保持土地承包关系稳定并长久不变；推进城镇和农村土地开发利用，加快农村宅基地和集体建设用地使用权确权登记颁证工作；抓好农村综合产权交易平台建设，强化农业保险保障作用。统筹推进司法、户籍、医疗等制度改革。提升对外开放水平。深入推进“一招四引”，大力引进平台型、基地型、龙头型、集群型企业和项目，实际利用境内外资金增长5%以上。积极融入“一带一路”、中原城市群建设等国家战略，提升中欧班列（新乡号）运营水平，鼓励我市优势企业走出去。加强异地商协会建设。开展“回归工程”，吸引在外兴办企业的新乡籍人士回乡创业。全面落实“一招四引”优惠政策，对本地企业新建、扩建的，按招商引资项目同等对待。扩大文化、教育、旅游、体育、卫生等领域开放。

　　（八）切实保障和改善民生。全面完成省市承诺办理的实事。优先发展教育事业。落实《新乡市中小学校幼儿园规划建设条例》，推进第三期学前教育行动计划，新建、改扩建公办幼儿园28所；完成卫辉市、延津县、长垣县义务教育发展基本均衡县创建任务，改造农村学校114所；加快普及高中阶段教育步伐，支持驻新高校发展，完善职业教育体系，深化产教融合、校企合作；支持和规范社会力量兴办教育；改善贫困地区义务教育办学条件，努力让每个孩子享有公平而有质量的教育。持续提高就业质量。加强政策扶持、创业培训，促进高校毕业生、就业困难人员、退役军人等重点群体多渠道就业创业，推动农村劳动力转移就业，妥善做好化解过剩产能和处置“僵尸企业”过程中下岗分流职工再就业工作，城镇新增就业7.35万人，城镇失业人员再就业1.6万人，新增农村劳动力转移就业2.8万人。健全社会保障体系。完善城乡统一的居民养老保险、基本医疗保险、大病保险、困难群众大病补充医疗保险和医疗救助等制度，扩大各项社会保险覆盖面，推进企业社会保险扩面征缴。开展健康养老示范工程，发展居家、社区和互助式养老，推进多种形式的医养结合。健全农村留守儿童、妇女、残疾人康复救助等服务体系。推进健康新乡建设。深化城市公立医院改革，推进分级诊疗、家庭医生医疗服务和医联体建设。加快中心医院东区医院、儿童医院、平原医院等重点项目建设。新建、改扩建一批县级医院、妇幼保健和疾病防控机构，提升基层医疗服务水平。启动实施中医药传承创新工程，发展中药材种植产业，重振百泉药交会品牌。加快平原体育中心建设，完善基层体育设施，开展全民健身活动，增强人民体质。推动文化发展繁荣。践行社会主义核心价值体系，开展群众性精神文明创建活动，推进“八大环境”建设，争创国家信用试点市。加强基层公共文化设施建设，提升文化惠民工程效能。制订文化产业发展规划，繁荣文艺创作，谋划实施一批文化产业园、文化产业项目和文化标志性工程。实施最严格的文物保护制度。加强网络建设治理，营造清朗网络空间。推动社会治理创新。加强和完善城乡社区治理。完善信访工作机制，加强诉调对接，解决好群众合理合法的利益诉求。推进应急体系建设，提升防灾减灾救灾能力。开展扫黑除恶专项斗争，严厉打击各类违法犯罪活动。支持群团组织更好开展工作。落实好党的民族、宗教和侨务政策。加强人防设施保护管理，深入开展双拥共建，支持国防和军队建设。统筹做好统计审计、气象地震、史志档案、新闻出版等工作，促进各项事业全面进步。

　　（九）强化政府自身建设。坚持以全面从严治党带动全面从严治政，不断提高执政能力和水平，建设人民满意政府。提高政治站位。树牢“四个意识”，坚定“两个维护”，不折不扣贯彻执行党中央国务院、省委省政府和市委的各项决策部署，确保令行禁止、政令畅通。坚定“四个自信”，准确把握习近平新时代中国特色社会主义思想内涵，紧密结合实际，创造性开展工作。全面依法行政。坚持依宪施政、依法行政，自觉接受市人大及其常委会的法律监督、工作监督和市政协的民主监督，高度重视公众监督和舆论监督，加强审计监督，确保各项工作在法律、法规框架内开展。严格规范政府行为，确保公正文明执法。坚持科学、民主、依法决策，凡涉及公众利益的重大事项，都要深入听取各方意见包括批评意见。加强作风建设。坚持“不忘初心、牢记使命”，推进“两学一做”学习教育常态化制度化。结合“敢转争”实践活动，聚焦项目建设、营商环境等重点领域，严厉查处各类懒政、怠政、庸政行为，推进行政效能提升、工作作风转变。各级各部门要站位全局，不上交矛盾、不推诿扯皮，以敬畏之心、带着责任对待工作和群众。完善正向激励、考核评价、容错纠错机制，倡树勇于担当、踏实做事、不谋私利的良好政风。坚持廉洁从政。深入落实中央八项规定精神，坚决防止“四风”变异反弹。支持纪检监察及司法机关严肃查处腐败案件，形成不敢腐的强大震慑；加强各项制度建设，扎牢不能腐的笼子；坚持廉政教育，筑牢不想腐的防线，切实做到干部清正、政府清廉、政治清明。

　　各位代表！新时代要有新气象，新征程要有新作为。让我们紧密团结在以习近平同志为核心的党中央周围，在省委、省政府和市委的坚强领导下，不忘初心、牢记使命、锐意进取，努力为中原更加出彩增光添彩，为决胜全面建成小康社会、建设富强民主文明和谐美丽的社会主义现代化强市而努力奋斗！
